# The 10 kDa domain of human erythrocyte protein 4.1 binds the *Plasmodium falciparum *EBA-181 protein

**DOI:** 10.1186/1475-2875-5-100

**Published:** 2006-11-06

**Authors:** Roberto Lanzillotti, Theresa L Coetzer

**Affiliations:** 1 Department of Molecular Medicine and Haematology, National Health Laboratory Service, School of Pathology, University of the Witwatersrand, Parktown, Johannesburg, 2193, South Africa

## Abstract

**Background:**

Erythrocyte invasion by *Plasmodium falciparum *parasites represents a key mechanism during malaria pathogenesis. Erythrocyte binding antigen-181 (EBA-181) is an important invasion protein, which mediates a unique host cell entry pathway. A novel interaction between EBA-181 and human erythrocyte membrane protein 4.1 (4.1R) was recently demonstrated using phage display technology. In the current study, recombinant proteins were utilized to define and characterize the precise molecular interaction between the two proteins.

**Methods:**

4.1R structural domains (30, 16, 10 and 22 kDa domain) and the 4.1R binding region in EBA-181 were synthesized in specific *Escherichia coli *strains as recombinant proteins and purified using magnetic bead technology. Recombinant proteins were subsequently used in blot-overlay and histidine pull-down assays to determine the binding domain in 4.1R.

**Results:**

Blot overlay and histidine pull-down experiments revealed specific interaction between the 10 kDa domain of 4.1R and EBA-181. Binding was concentration dependent as well as saturable and was abolished by heat denaturation of 4.1R.

**Conclusion:**

The interaction of EBA-181 with the highly conserved 10 kDa domain of 4.1R provides new insight into the molecular mechanisms utilized by *P. falciparum *during erythrocyte entry. The results highlight the potential multifunctional role of malaria invasion proteins, which may contribute to the success of the pathogenic stage of the parasite's life cycle.

## Background

Malaria is caused by a group of infectious protozoan parasites that alters the physiological functioning and cellular biology of erythrocytes. *Plasmodium falciparum *is the best-studied species and is the commonest, most virulent and principal cause of the majority of infections and deaths worldwide. Since the completion of the parasite's genome sequence [1], a wealth of knowledge has been generated through proteomic and genomic studies. High-throughput screening [2,3], protein identification technology such as mass spectrometry [4-7] and gene knockdown/knockout technology [8-13] are some of the methods that have been used to decipher complex molecular processes governing the life cycle of *P. falciparum*.

The invasion of erythrocytes by *P. falciparum *initiates the pathogenic phase of the life cycle and is essential for parasite survival and progression of clinical malaria [14]. Invasion is a complex process involving a series of molecular interactions between *P. falciparum *merozoites and erythrocyte membrane receptors. Mediators of invasion have been identified on the merozoite surface and in organelles of the apical complex, namely rhoptries, micronemes and dense granules. However, the precise functioning of these proteins and mechanisms governing the entry process are poorly understood [15,16].

A family of erythrocyte Duffy binding-like (DBL) proteins, located within the micronemes, plays a crucial role in the binding of merozoites to the host cell. In *P. falciparum*, six homologous DBL genes have been found, including erythrocyte binding antigen-175 (EBA-175), EBA-181 (JESEBL), EBA-140 (BAEBL), EBA-165 (PEBL), MAEBL and erythrocyte binding ligand-like 1 protein. These genes encode several domains including two cysteine-rich regions. These regions comprise one or two copies of an amino DBL domain which defines the erythrocyte-binding region and a C-terminal domain of unknown function [17]. Each protein encodes unique receptor specificity, which allows *P. falciparum *to diversify the number of invasion pathways [18].

EBA-181 has been identified on chromosome 1 of the *P. falciparum *genome. The protein is expressed in merozoites as well as schizonts and evidence suggests that this ligand plays a role during erythrocyte invasion [19,20]. Eight polymorphisms have been found in the erythrocyte binding domains, which have been hypothesized to promote survival advantage in parasites exposed to genetically diverse human populations [21].

Recent work utilizing *P. falciparum *phage display libraries has identified a protein-protein interaction between erythrocyte membrane protein 4.1 (4.1R) and EBA-181 [22,23]. 4.1R is an important component of the erythrocyte skeleton, which provides mechanical strength to the membrane through vertical interaction with glycophorin C/D and horizontal association with spectrin and actin [24]. The interaction of 4.1R with EBA-181 suggests a new role for this protein at the host-parasite interface. In this study, recombinant proteins were used to map the domain in 4.1R responsible for binding to the parasite protein.

## Methods

### Preparation of recombinant proteins

**Figure 1:**
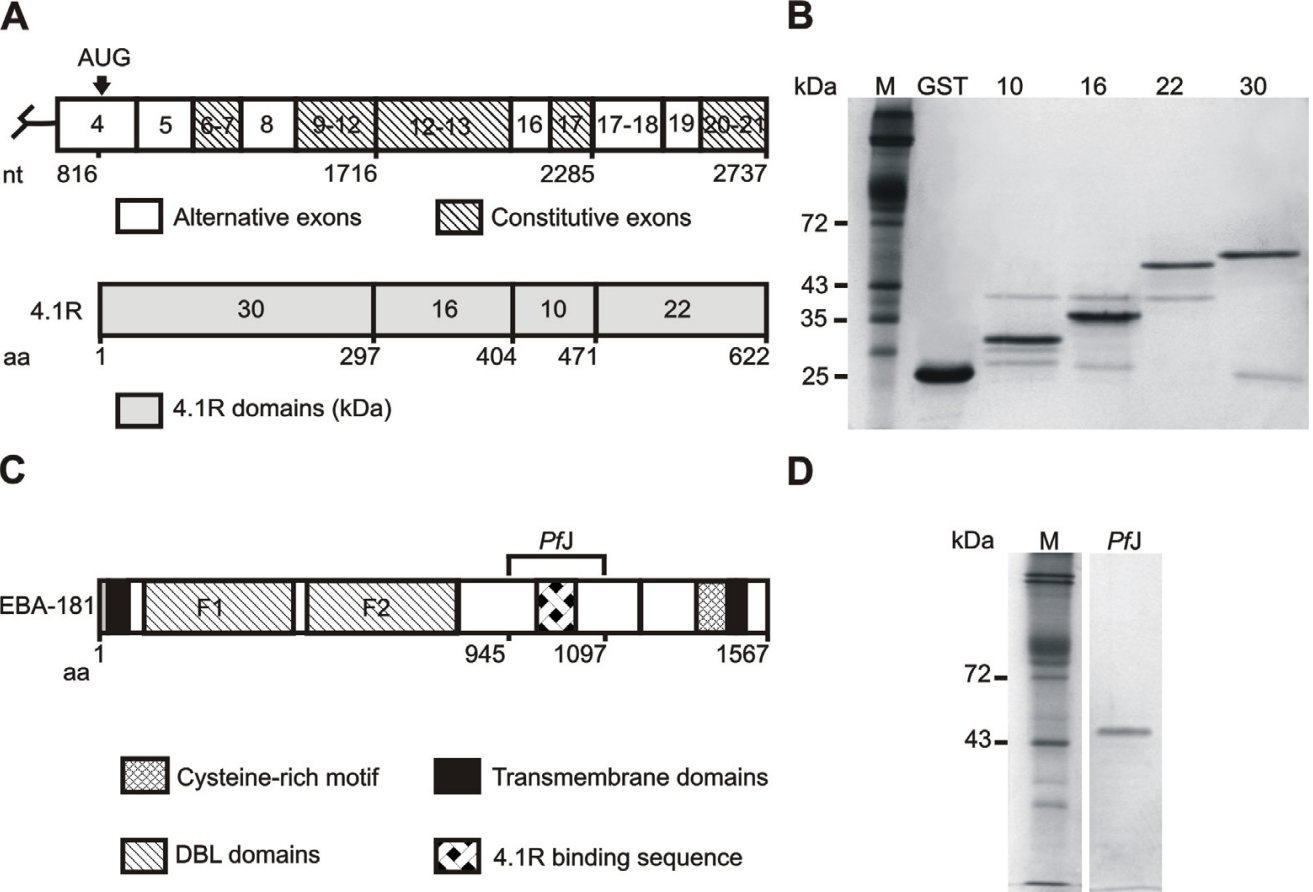
**Schematic representation of 4.1R and EBA-181 and purification of the respective fusion proteins**. (**A**) Schematic of 4.1 cDNA showing alternative and constitutive exons. The erythrocyte translation initiation site is indicated at nucleotide (nt) position 816 in exon 4 with the coding sequence extending to position 2737. The corresponding full-length 80 kDa-4.1R molecule is shown below the cDNA. Amino acid (aa) residue numbers depict the relative locations of the four 4.1R structural domains [26-28]. The 30 kDa domain (aa 1–297) is located at the N-terminal end of the protein and corresponds to nt 816–1716. The 16 kDa and 10 kDa domains are encoded by nt 1717–2285 (aa 298–471), with the 22 kDa region situated at the C-terminal end. (**B**) GST-4.1R fusion domains were expressed and purified using glutathione magnetic affinity beads. Approximately 1–2 μg of total protein was resolved by 12% SDS-PAGE. The protein samples are erythrocyte membrane marker M (lane 1), GST control (lane 2), GST-10 kDa (lane 3), GST-16 kDa (lane 4), GST-22 kDa (lane 5) and GST-30 kDa (lane 6). (**C**) Schematic of the full-length *P. falciparum *invasion protein EBA-181 [43], accession number: PFA0125c. Amino acid (aa) numbers underneath the schematic demarcate the various domains in the protein. The molecule comprises two DBL domains (denoted F1 and F2) which define erythrocyte specificity, as well as two transmembrane regions and a C-terminal cysteine-rich motif. The relative position of the EBA-181 fragment (*Pf*J) used in this study is shown (aa 945–1097; expected size 25 kDa). (**D**) 6His-*Pf*J was expressed and purified from crude *E. coli *lysates using magnetic nickel beads and resolved by SDS-PAGE: M, erythrocyte membrane marker (lane 1) and purified *Pf*J (lane 2), which resolves at ~50 kDa.

Total RNA was extracted from human reticulocytes [25] and the four structural domain sequences of 4.1R (30 kDa, 16 kDa, 10 kDa and 22/24 kDa, Figure 1A) [26-28] were reverse transcribed using AMV reverse transcriptase (Promega, USA). Primers flanking the relevant domains were designed and restriction sites for *Bam*H I or *Eco*R I and *Xho *I appended at the 5' and 3' ends respectively (Table 1). The 4.1R cDNA was amplified by polymerase chain reaction (PCR), digested with appropriate restriction enzymes and subcloned into the pGEX-4T-2 protein expression vector (Amersham Biosciences, UK) according to standard methods. The reading frame and PCR product sequences were verified using automated DNA sequencing. 4.1R vector constructs were transfected into Rosetta™ 2 (DE3) *Escherichia coli *(*E. coli*) cells (Novagen, USA) and expression of glutathione-S-transferase (GST)-tagged polypeptides induced with either 1 mM isopropyl β-D-thiogalactopyranoside (IPTG) (Invitrogen, USA) or Overnight Express™ Autoinduction System (Novagen, USA). GST-tagged proteins were purified from *E. coli *extracts using MagneGST™ agarose beads (Promega, USA).

**Table 1:** Oligonucleotide primers

**Domain**	**PCR primer (5'-3')**	**RE**
10 kDa	F: catg*ggatcc*tggaagaaaaagagagaaag	*Bam*H I
	R: catg*ctcgag*tcagggtgagtgagtggataag	*Xho *I
16 kDa	F: catg*ggatcc*tttcgatacagtggccggact	*Bam*H I
	R: catg*ctcgag*tcatgcttctgtgggctctggct	*Xho *I
22 kDa	F: catg*ggatcc*ttccgaactcttaacatcaatgggcaaa	*Bam*H I
	R: catg*ctcgag*tcactcatcagcaatctcggtctcc	*Xho *I
30 kDa	F: catg*gaattc*atgcactgcaaggtttctttgt	*Eco*R I
	R: catg*ctcgag*tcatttggatcctagcgcaag	*Xho *I
*Pf*J	F: catgcatg*catatg*cctgaagtagttccacaagaa	*Nde *I
	R: catg*ggatcc*ttatgcactttcacctccccc	*Bam*H I

Forward (F) and reverse (R) primer sequences were designed for amplifying the different human 4.1R domain sequences and *P. falciparum *4.1R binding sequence (*Pf*J) in EBA-181. Endonuclease recognition sequences are underlined and the corresponding restriction enzyme (RE) indicated.

A histidine (6His)-tagged peptide (denoted *Pf*J) encompassing the 4.1R binding region in EBA-181 was prepared (Figure 1C). *P. falciparum *was cultured *in vitro *by the method of Trager and Jensen [29] and genomic DNA extracted using phenol-chloroform and ethanol precipitation. The EBA-181 binding sequence was amplified from parasite DNA by PCR, using primers containing 5' *Nde *I and 3' *Bam*H I recognition sequences (Table 1). *Pf*J was subcloned into pET15b vector (Novagen, USA), the insert sequenced and the vector construct transfected into BL21-CodonPlus^® ^(DE3) RIL competent cells (Stratagene, USA). 6His-*Pf*J was induced using 1 mM IPTG and purified from *E. coli *extracts using His-Select™ magnetic agarose beads (Sigma, USA).

Recombinant proteins were dialyzed against PBS (10 mM Na_2_HPO_4_, 1.5 mM KH_2_PO_4_, 137 mM NaCl, 2.7 mM KCl, pH 7.4) in a Slide-A-Lyzer MINI dialysis unit (Pierce, USA) for 2 hrs at 8°C. Protein concentrations were determined spectrophotometrically at 595 nm using the Coomassie Plus^® ^Protein Assay Reagent Kit (Pierce, USA). Protein aliquots were analyzed by 12% sodium dodecyl sulphate polyacrylamide gel electrophoresis (SDS-PAGE) and samples stored at -20°C in a final concentration of 1 mM Pefablock^® ^SC (Roche, Germany).

### Blot overlay assays

Blot overlay assays were used for studying protein-protein interactions between recombinant proteins. The objective was to map the interaction between 6His-*Pf*J and GST-4.1R domains.

Approximately 5 μg of GST, GST-4.1R structural domains and 6His-parasite protein were spotted onto Hybond C nitrocellulose membrane strips (Amersham, UK). Additional binding sites were blocked in 5% BSA in TBS (0.05 M Tris-HCl, 0.9% NaCl, pH 7.5) for 1 hr and the membranes overlaid with 0.5–1 μg of either purified 4.1R [30] or appropriate recombinant domain for 2 hrs. GST and the 4.1R domains served as negative and positive controls respectively. The strips were washed twice for 1 minute each in TBS and the bound protein fixed to the membrane with 0.5% (v/v) formaldehyde in TBS for 20 minutes. Membranes were washed for 15 minutes in TBS and probed with 1:1,000 rabbit anti-4.1R antibody (St. Elizabeth's Medical Centre, Boston, USA) for at least 1 hr. Interactions were detected with 1:1,000 goat anti-rabbit IgG peroxidase conjugated antibody (Roche, Germany) using Supersignal^® ^West Pico Chemiluminescent substrate (Pierce, USA).

### Pull-down assays

Pull-down assays were used to verify the interaction between *P. falciparum *protein and the specific 4.1R domain. Furthermore, the dependence of the interaction on concentration was determined by densitometric scanning of 12% SDS-PAGE gels.

Approximately 700 ng 6His-*Pf*J was coupled to 30 μl aliquots of His-Select™ magnetic beads. Various amounts of specific GST-4.1R domain, ranging from 150 ng to 2 μg, as well as 0.5 μg, 5 μg and 10 μg heat denatured protein were added to the beads and incubated for 1 hr at room temperature. The denatured samples served as a control to correct for non-specific binding. The beads were rinsed with TBS and the protein complexes eluted using 1% SDS in TBS for 10 minutes. Samples were analyzed by SDS-PAGE and 12% polyacrylamide gels scanned using a Hoefer transmittance/reflectance scanning densitometer (Hoefer, USA). Areas under the peaks were compared to standard GST-10 kDa and 6His-*Pf*J curves and the ratio of bound protein (μg) to parasite protein (μg) determined. A curve was plotted against the amount of 4.1R domain to determine whether the interaction was saturable.

## Results and discussion

### Recombinant proteins

Rosetta 2 (DE3) cells were transformed with the different 4.1R domain constructs and induced with 1 mM IPTG. Under these conditions, the expressed 10 kDa, 16 kDa and 22 kDa recombinant proteins were soluble, while the GST-tagged 30 kDa protein was completely insoluble. The use of the Overnight Express™ Autoinduction system significantly improved the yield of soluble GST-30 kDa. 0.5–1 μg of purified protein was obtained from 1 ml IPTG-induced culture volumes or 25 ml Overnight Express cultures. Scanning densitometry of a 12% SDS polyacrylamide gel revealed that purified proteins were more than 85% pure (Figure 1B). A Western blot was performed to confirm the identity of GST-tagged 4.1R domains (data not shown). 6His-*Pf*J was expressed predominantly as a soluble protein as evidenced by SDS-PAGE analysis and its identity confirmed with Western blot analysis (data not shown). Approximately 0.8 μg of protein was obtained from 1 ml *E. coli *culture. 6His-*Pf*J was purified to more than 95% homogeneity as determined by scanning densitometry. Polyacrylamide gels indicated that *Pf*J migrated as an apparent dimer (~50 kDa) under SDS-PAGE conditions (Figure 1D). This is most likely attributed to the high proportion of charged amino acids in *Pf*J, which presumably resulted in atypical binding of SDS molecules leading to aberrant and unpredictable migration patterns on denaturing polyacrylamide gels [31].

### 6His-PfJ binds the 10 kDa domain of 4.1R

**Figure 2:**
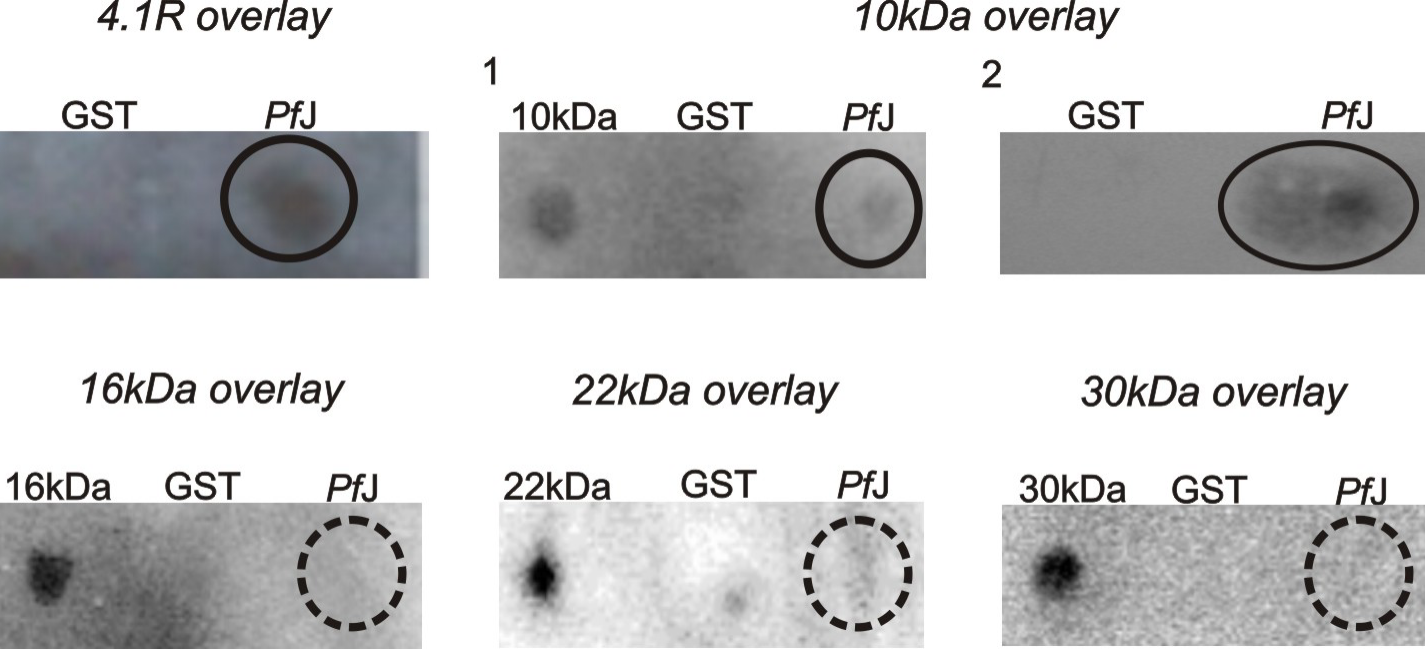
**Blot overlay assays of 6His-*Pf*J with native 4.1R and GST-tagged 4.1R domains**. The headings above each Western blot represent the proteins spotted on the nitrocellulose membrane. Glutathione-S-transferase (GST) and the relevant 4.1R domain served as negative and positive controls respectively. The membranes were overlaid with native 4.1R (*4.1R overlay*) and GST-tagged domains (*10 kDa*, *16 kDa *and *30 kDa overlays*) and binding detected with polyclonal rabbit anti-4.1R antibody using a chemiluminescent substrate. All the 4.1R fragments were detectable using the primary polyclonal antibody as demonstrated by the positive controls on the overlays. The *4.1R overlay *indicates that 6His-*Pf*J binds native 4.1R. Two independent experiments (**1 **and **2**) revealed specific interaction between 6His-*Pf*J and GST-10 kDa.

**Figure 3:**
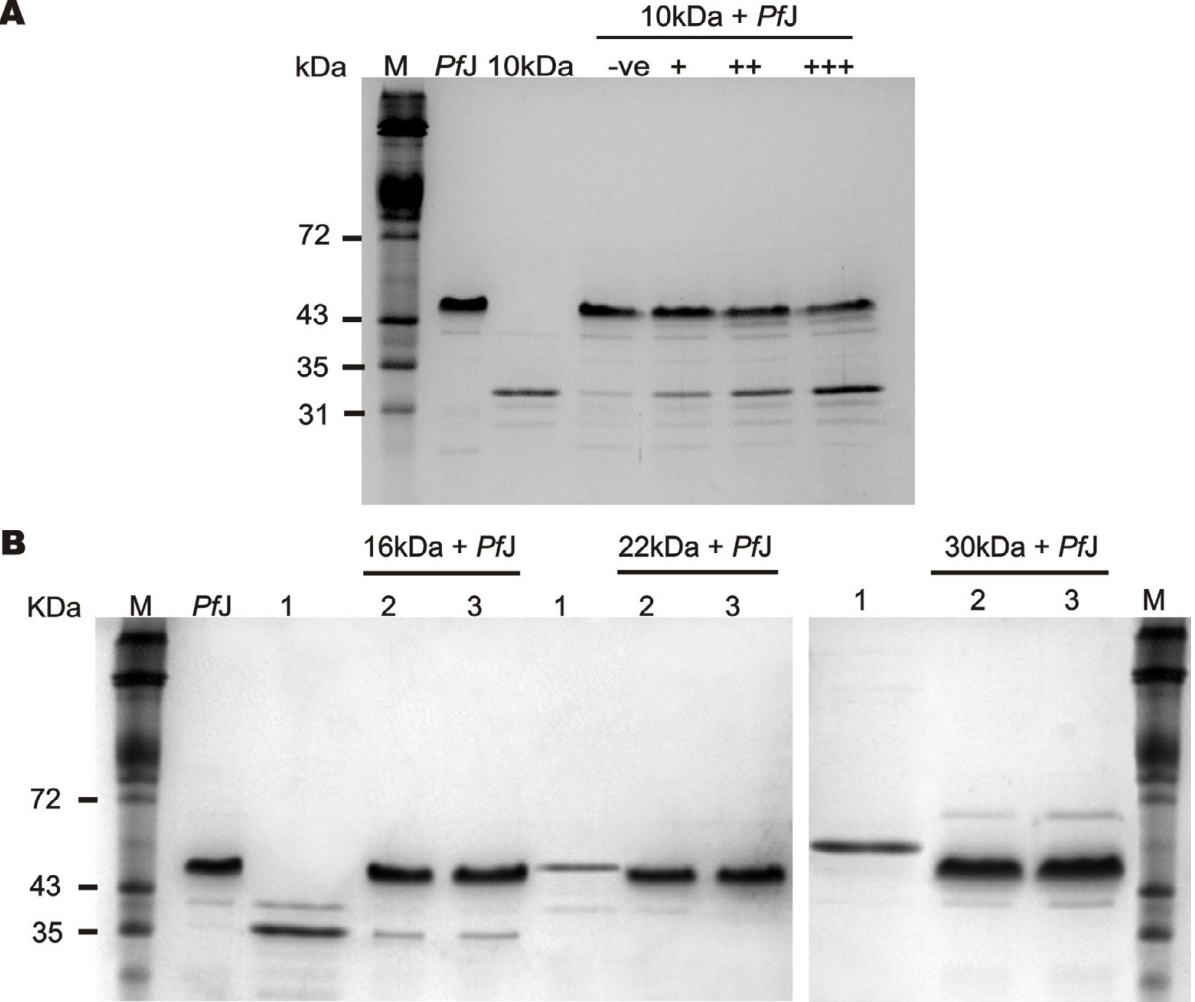
**Histidine pull-down assays of GST-4.1R recombinant proteins**. **A**) 6His-*Pf*J bound specifically to GST-10 kDa in a histidine pull-down assay. The interaction was dose-dependent and reliant on native GST-10 kDa protein conformation. **M**, erythrocyte membrane marker; **-ve**, negative control pull-down using 5 μg heat denatured GST-10 kDa and **+**, assays indicating the dose-dependent nature of the interaction (+/++/+++ ≈ 150 ng/400 ng/800 ng GST-10 kDa respectively). **B**) 6His-*Pf*J (~800 ng) did not bind specifically to ~6 μg GST-16, 22 and 30 kDa proteins. **1**, purified GST-4.1R domain; **2**, histidine pull-down using heat denatured recombinant protein and **3**, pull-down using non-denatured protein.

Recombinant proteins were used in blot overlay and histidine pull-down assays to determine the binding site in 4.1R specific for 6His-*Pf*J. Blot overlay assays showed that 6His-*Pf*J bound specifically to the GST-10 kDa domain as well as native 4.1R (Figure 2). The recombinant parasite protein did not bind GST nor the 16 kDa, 22 kDa and 30 kDa GST-tagged domains. To confirm the interaction between GST-4.1R fragments and 6His-*Pf*J, histidine pull-down assays were performed. In agreement with the blot overlay data, GST-10 kDa bound specifically to 6His-*Pf*J in a dose-dependent manner (Figure 3A). The interaction was abolished by heat denaturation of the 10 kDa domain. Histidine pull-down assays were also performed with GST-16, 22 and 30 kDa proteins and 6His-*Pf*J. As shown in figure 3B, no binding was detectable in the 30 and 22 kDa reactions. The GST-16 kDa pull-down assay revealed some binding with 6His-*Pf*J, but this was non-specific as the interaction was comparable to heat-denatured 4.1R domain.

**Figure 4:**
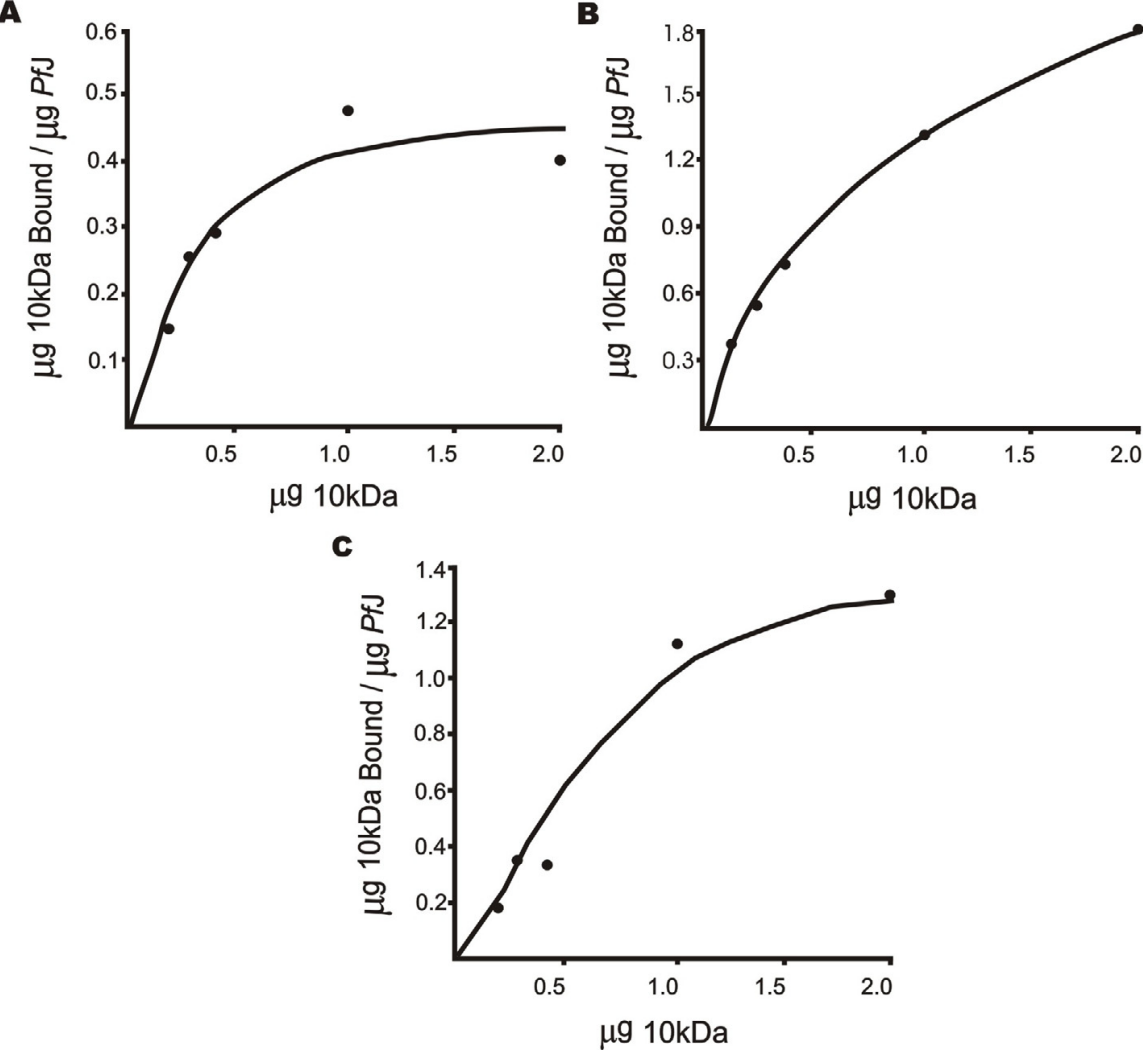
**GST-10 kDa/6His-*Pf*J solution binding assays**. GST-10 kDa (200, 300, 400, 1000 and 2000 ng) bound to ~700 ng 6His-*Pf*J on His-Select™ magnetic beads in a concentration dependent manner (**A-C**). Protein complexes were resolved on 12% Laemmli gels and analyzed with scanning densitometry. Non-specific binding of heat denatured GST-10 kDa (54 ng/μg *Pf*J) was subtracted from the amount of bound protein. **A**, **B **and **C **represent independent experiments using two different protein preparations.

To determine whether the interaction between 6His-*Pf*J and GST-10 kDa was saturable, the parasite protein was incubated with a concentration range of 10 kDa domain. Scanning densitometry of the polyacrylamide gels revealed that the interaction was concentration dependent and saturable in three independent experiments using different protein preparations (Figure 4A–C). To compensate for non-specific binding, 54 ng heat denatured GST-10 kDa per μg 6His-*Pf*J was subtracted from the amount of bound protein. This value was determined by averaging the non-specific binding displayed by 0.5 μg and 10 μg of heat denatured 4.1R domain, which was 51 and 58 ng/μg *Pf*J respectively. This result demonstrated that 6His-*Pf*J bound specifically to GST-10 kDa. To allow semi-quantitative assessment of the interaction, a rough estimate of the dissociation constant (Kd) was obtained by linear transformations of the three curves which gave an average Kd of 745 nM.

### *P. falciparum *and erythrocyte invasion proteins

The *in vitro *association of EBA-181 with an underlying erythrocyte skeletal protein has important implications for *P. falciparum *invasion. 4.1R is a critical component of the erythrocyte skeleton. It functions by stabilizing horizontal protein interactions and links the underlying skeleton to the lipid bilayer [32]. The conserved 10 kDa domain of 4.1R interacts with a number of key proteins, and represents a pivotal point in the control of erythrocyte membrane integrity. The domain facilitates the interaction between spectrin and actin, and in so doing maintains the deformability of the erythrocyte [24]. The 10 kDa region has been shown to bind and regulate myosin activity [33]. Fowler and colleagues [34] proposed that erythrocyte myosin could function together with actin and its associated proteins in an actomyosin contractile apparatus, raising the possibility of an ATP-dependent process responsible for regulating shape transformations in human erythrocytes. Cibert and colleagues [35] explored a potential role for actomyosin complexes in the restoration of erythrocyte membrane skeletons, damaged by mechanical or chemical stress. Their model proposes that upon disruption of the skeletal network, cytosolic myosin is relocated to the erythrocyte bilayer where formation of an actomyosin complex initiates repair of the relevant area. The repair process was suggested to involve a stable linkage of actin protofilaments by myosin filaments around the area of damage. The disruption of linkages between 4.1R, spectrin, actin and myosin, therefore, has serious consequences for the stability and repair of erythrocyte membranes.

Damage inflicted by invading merozoites to the erythrocyte's protein and lipid bilayer, may initiate actomyosin repair processes. This would subsequently lead to the restoration of the damaged areas and provide a barrier for merozoite entry. *P. falciparum*, having sustained a long-standing association with human hosts, evolved complex evolutionary adaptations to ensure its own survival. It may be conceivable that specific *P. falciparum *proteins evolved to subvert host membrane repair. The interaction between EBA-181 and the 10 kDa domain of 4.1R may be such a mechanism, whereby EBA-181 blocks the binding of myosin to 4.1R. This prevents the activation of actomyosin repair machinery. As a result, the parasite modulates host cellular processes to guarantee successful invasion.

Binding of the *P. falciparum *invasion protein, EBA-181, to 4.1R may thus facilitate invasion and prevent repair of the damage to the erythrocyte membrane, prior to parasite entry. Based on published findings from other groups and the EBA-181/4.1R interaction described in this work, the following speculative model of *P. falciparum *erythrocyte entry is proposed: Following initial attachment to the host cell surface, the merozoite reorients to bring its apical end in close contact with the host membrane [16]. An increase in the parasite's intracellular calcium concentration signals the secretion of the microneme and rhoptry contents onto the erythrocyte surface [36]. With the aid of a C-terminal transmembrane domain, DBL proteins are translocated from the micronemes onto the merozoite surface, where the DBL binding domains interact with erythrocyte receptors [37]. However, it is possible that full-length invasion proteins or modified forms [38,39] interact with the erythrocyte skeleton. This would be made possible through the action of lipases and proteases from the merozoite's apical organelles. These molecules are responsible for initiating structural changes in the erythrocyte membrane including skeletal and surface modifications [31,40-42]. It is therefore likely that the cleavage of proteins and phospholipids on the erythrocyte membrane during invasion will facilitate the transient access of EBA-181 to 4.1R.

## Conclusion

The intimate interaction between *P. falciparum *and the erythrocyte membrane has provided fascinating insight into the cell biology of the malaria parasite. The interaction of EBA-181 with the highly conserved 10 kDa domain in 4.1R highlights the complex role of microneme invasion proteins. These proteins may potentially serve multiple functions to mediate parasite entry into the erythrocyte, including: 1) the initiation of merozoite invasion by binding specific erythrocyte membrane receptors; 2) the destabilization of spectrin-actin interactions and framework of the erythrocyte skeleton as a consequence of binding to 4.1R, and 3) EBA-181 may inhibit potential host membrane repair pathways.

## Authors' contributions

RL prepared the recombinant proteins, carried out the molecular interaction studies and drafted the manuscript. TLC was involved in the design and coordination of the study, and assisted in drafting the manuscript. All authors read and approved the final manuscript.
